# Human Circovirus in Patients with Hepatitis, Hong Kong 

**DOI:** 10.3201/eid3012.241114

**Published:** 2024-12

**Authors:** Shusheng Wu, Cyril Chik-Yan Yip, Jianwen Situ, Zhiyu Li, Stanley Siu-Fung Ho, Jianpiao Cai, Jane Hau-Ching Poon, Nicholas Foo-Siong Chew, Jonathan Daniel Ip, Tom Wai-Hin Chung, Kelvin Hei-Yeung Chiu, Anna Jinxia Zhang, Estie Hon-Kiu Shun, James Yiu-Hung Tsoi, Jade Lee-Lee Teng, David Christopher Lung, Kelvin Kai-Wang To, Vincent Chi-Chung Cheng, Irene Oi-Lin Ng, Kwok-Yung Yuen, Siddharth Sridhar

**Affiliations:** The University of Hong Kong, Hong Kong, China (S. Wu, C.C.-Y. Yip, J. Situ, Z. Li, S.S.-F. Ho, J. Cai, J.H.-C. Poon, N.F.-S. Chew, J.D. Ip, A.J. Zhang, J.Y.-H. Tsoi, J.L.-L. Teng, K.K.-W. To, V.C.-C. Cheng, I.O.-L. Ng, K.-Y. Yuen, S. Sridhar); Queen Mary Hospital, Hong Kong (T.W.-H. Chung, K.H.-Y. Chiu, V.C.-C. Cheng); Queen Elizabeth Hospital, Hong Kong (D.C. Lung); Hong Kong Children's Hospital, Hong Kong (D.C. Lung); Centre for Virology, Vaccinology and Therapeutics, Hong Kong (E.H.-K. Shun, K.K.-W. To, K.Y. Yuen)

**Keywords:** hepatitis viruses, circovirus, viruses, infectious mononucleosis, chronic hepatitis, Hong Kong, China

## Abstract

*Circovirus human* is a new viral species that includes the human circovirus (HCirV), which has been linked to hepatitis in immunocompromised persons. We investigated prevalence of HCirV infection in 278 patients with hepatitis and 184 asymptomatic persons using real-time PCR and sequencing assays. HCirV viremia and sequences were found in 8 (2.9%) hepatitis patients and no asymptomatic patients. Alternate causes of hepatitis (hepatitis E and cholangitis) were clearly identifiable in 2 HCirV-infected patients. HCirV could not be ruled out as a contributor to hepatitis in the remaining 6 patients, 4 of whom were immunocompromised. Persistent infections were documented in 3 patients, but only 1 had relapsing hepatitis. One HCirV patient displayed symptoms of an infectious mononucleosis-like syndrome. Isolates clustered with known HCirV strains from France and China. HCirV-derived virus-like particles bound to PLC/PRF/5 and Hep-G2 human hepatoma cells but not to lung epithelial cells, indicating hepatic tropism.

Hepatitis is a common entity; severity can range from subclinical biochemical transaminitis to acute liver failure. Common etiologies of hepatitis include viral infections, adverse drug reactions, alcohol, autoimmune disorders, metabolic diseases, and biliary pathologies. However, unexplained hepatitis is still frequently encountered in clinical practice. Studies in a variety of practice settings estimate that 11%–35% of acute liver failure cases lack a clear etiology (*1*). This proportion is higher in patients with mild hepatitis, for whom exhaustive laboratory investigations are usually not performed. Therefore, conceivably, some causes of infectious hepatitis remain unknown. A striking example of this possibility was the recent multinational outbreak of pediatric hepatitis for which the best current hypothesis is a co-infection of adeno-associated virus 2 and adenovirus type 41 in children with genetic predispositions (*2*). Another example is rat hepatitis E virus, previously disregarded as a human pathogen but now identified as an endemic hepatitis agent in China and Spain (*3*–*5*).

*Circovirus human* is a recently discovered species of the family Circoviridae independently reported in France and China (*6*–*8*). This species includes human circovirus (HCirV). In China, HCirV was first reported in an intravenous drug user with HIV infection (*6*). A follow-up study identified a second HCirV patient, also an intravenous drug user with HIV and hepatitis C virus (HCV) co-infection (*9*). Sparse clinical details were available for those 2 patients. Around the same time, researchers in France using metagenomic next-generation sequencing identified HCirV infection in a heart–lung transplant recipient with unexplained hepatitis (*7*). HCirV peripheral blood virus loads correlated with derangements in liver function tests (LFTs). Those researchers also demonstrated HCirV mRNA in liver tissue by in situ hybridization indicating virus tropism for hepatocytes (*7*).

In this study, we evaluated the role of HCirV as a cause of viral hepatitis by comparing HCirV infection prevalence in hepatitis cases and asymptomatic controls. We described the clinical profiles and genomic epidemiology of HCirV infection cases. Finally, we expressed HCirV virus-like particles (VLPs) to probe binding of HCirV to human hepatic cell lines.

## Materials and Methods

### Study Setting, Patient Specimens, and Animal Samples

This retrospective observational study was conducted at the Queen Mary Hospital (QMH) Department of Microbiology in Hong Kong, China. The study was approved by the Institutional Review Board of the University of Hong Kong, China/Hospital Authority Hong Kong, China West Cluster (UW 23-330). The QMH microbiology laboratory provides viral hepatitis diagnostic services to acute hospitals and transplant services across Hong Kong. We retrieved plasma or serum samples from patients sent to the laboratory for diagnostic evaluation of viral hepatitis (hepatitis cases) during October 1, 2022–November 15, 2023. We assigned patients to the hepatitis cases group if alanine aminotransferase (ALT), aspartate aminotransferase (AST), alkaline phosphatase (ALP), or bilirubin were elevated above respective reference ranges (Appendix Table 1). We excluded persons with isolated elevated ALP if their gamma-glutamyl transpeptidase levels were within reference ranges (indicating extrahepatic source). Similarly, we excluded cases with isolated unconjugated hyperbilirubinemia. Archived plasma or serum samples from persons with unremarkable LFTs formed a control group; blood samples from those persons were sent to the QMH microbiology laboratory for organ donation evaluation or cytomegalovirus (CMV) reactivation monitoring. We retrieved patient clinical notes, imaging findings, and diagnostic test results from electronic patient records. As described in our previous study (*5*), we defined patients as immunosuppressed if they met any of the following criteria at time of blood taking: active hematological malignancy, history of solid organ or hematopoietic stem cell transplantation, receiving disease modifying antirheumatic drugs/marrow-suppressive chemotherapy/steroids (>0.5 mg/kg/d prednisolone-equivalent for >1 mo), or living with HIV infection with CD4 T-lymphocyte counts <200 cells/mm^3^.

HCirV PCR screening was conducted by staff blinded to group assignment. Patients were defined as infected with HCirV if they fulfilled both of the following criteria: HCirV PCR positive in serum or plasma with cycle threshold (Ct) <36 and at least a partial HCirV genome fragment sequenced from serum or plasma. The stringent requirement for sequenced isolates eliminated spurious cases of nonspecific PCR positivity because we did not have positive sample controls for PCR reactions at the outset of this study. Clinical records of HCirV-infected persons were assessed for hepatitis at time of blood taking. If hepatitis was present, clinical, imaging, and diagnostic investigation data were independently assessed by 2 experts blinded to study goals to determine the cause of hepatitis.

### Real-Time PCR, Sequencing, and Phylogenetic Analysis

The HCirV real-time PCR targets the *rep* gene and was designed based on published genomes of HCirV strains from France (Paris strain) and China (YN09/J030 and YN09/347 strains) (GenBank accession nos. ON677309, ON226770, and OP744467) (Appendix Table 2). We determined performance characteristics including limit-of-detection, linearity, and analytical specificity of the assay as previously described (*10*,*11*) and obtained partial HCirV genomes by using Sanger sequencing. If the viral load was sufficiently high by Sanger sequencing, we attempted complete genome sequencing (Appendix Table 3). For phylogenetic analysis, we obtained complete genomes of the 65 exemplar circovirus species as listed by the International Committee on Taxonomy of Viruses as of September 9, 2024, from the National Center for Biotechnology Information (NCBI) GenBank database. We also included an additional 3 complete HCirV genomes (GenBank accession nos. OP744467.1, ON226770.2, and OR905605.1) and those of 4 strains of *Circovirus porcine 3* (PCV3; accession nos. MK095623, MK496297, MH277112, and MK496292). We downloaded 3 complete *Cyclovirus* genomes (accession nos. KT732787, KF031466, GQ404857) for use as an outgroup to root the phylogeny. 

We performed multiple sequence alignment of the complete genomes using MAFFT version 7.526 software (–genfapair–maxiterate 1000—adjustdirectionaccurately) (*12*) and added partial genome sequences to the alignment with MAFFT (–localpair–addfragments–adjustdirectionaccurately) (*13*). We removed sites with >20% gaps with TrimAl version 1.4.22 (-gt 0.2) (*14*) and visualized the resulting alignment in JalView (*15*). We inferred a maximum-likelihood phylogenetic tree using IQ-TREE, using the best-fit substitution model automatically selected by ModelFinder and MixtureFinder (*16*,*17*; H. Ren et al., unpub. data, https://www.biorxiv.org/content/10.1101/2024.03.20.586035v2). We assessed branch support using the Shimodaira-Hasegawa–like approximate likelihood ratio test with 10,000 bootstrap replicates. We visualized and annotated the final phylogenetic tree using TreeViewer (*18*).

### HCirV Peptide Expression, Characterization, and Immunoblots

The capsid (Cap) protein encoding the outer capsid of HCirV was expressed in *Escherichia coli*. Proteins were assessed using transmission electron microscopy and sodium dodecyl sulfate polyacrylamide gel electrophoresis (SDS-PAGE). Cap protein was used to raise hyperimmune polyvalent serum samples in mice and IgM/IgG immunoblots of human serum samples as previously described (*19*) (Appendix). 

### Cell-Binding Assay

We seeded human hepatoma cells (PLC/PRF/5, ATCC CRL-8024; Hep-G2, ATCC CRL-10741) and A549 lung epithelial cells (CCL-185) on glass coverslips in a 24-well plate. When confluence reached 80%, we washed cells with prechilled 1× phosphate-buffered saline (PBS). We incubated Purified Cap protein (10 µg/well) VLPs and control proteins with PLC/PRF/5, Hep-G2, and A549 cells for 1 hour at 4°C. After discarding supernatant, we extensively washed cells with cold PBS 3–4 times to remove unbound VLPs and fixed with 4% paraformaldehyde, then incubated with mouse anti-Cap protein polyvalent immune serum (1:2,00) diluted in 1% BSA. We used Alexa Fluor 488-conjugated anti-mouse IgG (ThermoFisher Scientific, https://www.thermofisher.com) as secondary antibody (1:2000). We counterstained nuclei at AntiFade mounting medium with DAPI (VectorLabs, https://www.vectorlabs.com). We examined slides using a fluorescence microscope and used ImageJ software (https://imagej.net/ij) to measure positive cells.

### Testing for Other Causes of Viral Hepatitis

We screened samples positive for HCirV DNA for other viral causes of hepatitis including hepatitis A virus, hepatitis B virus, HCV, hepatitis E virus (HEV), adenovirus, enterovirus, human herpesvirus (HHV) 6, HHV-7, CMV, HIV, and Epstein-Barr virus (EBV) using commercial or in-house–developed serologic and molecular assays (Appendix Table 4). Protocols for molecular assays have been published previously (*10*,*11*,*20*–*23*). We measured Torque teno virus (TTV) DNA using a published protocol (*24*).

### Statistical Analysis

We compared proportions of HCirV-positive cases and controls using χ^2^ or Fisher exact test, depending on the number of persons. To differentiate cholestatic from hepatocellular liver injury, we calculated the R factor as follows: (ALT value/ALT upper limit of normal) ÷ (ALP value/ALP upper limit of normal). R factor <2 corresponds to cholestatic injury, >2 to <5 represents a mixed pattern, and >5 corresponds to hepatocellular injury (*25*).

## Results

### Cases and Controls

We obtained serum or plasma samples from 278 hepatitis patients and 184 controls (Table 1). Immunosuppressed persons were well represented in both groups and accounted for more than half of all controls (Table 1). Degree of hepatitis was typically mild; most patients had anicteric hepatitis. A cause of hepatitis was identified by the clinical team in 123 (44.2%) cases; the remaining 155 (55.8%) were of unspecified etiology. For those with identified etiology, viral hepatitis (18.7%) and drug-induced liver injury (17.9%) were the most commonly diagnosed (Appendix Table 5).

**Table 1:** Clinical and demographic characteristics of hepatitis cases and asymptomatic controls in study of human circovirus in patients with hepatitis, Hong Kong

Characteristic	Hepatitis cases, n = 278	Controls, n = 184
Median age, y (IQR)	59 (46–69)	53; 38.8 – 61
Sex		
M	143 (51.4)	96 (52.2)
F	135 (48.6)	88 (47.8)
Immunosuppression	95 (34.2)	117 (63.6)
Hemopoietic stem cell transplant	24	32
Solid organ transplant	43	67
HIV	1	1
Hematological malignancy	14	14
Cancer/chemotherapy	8	0
Other	5	3
Median ALT, U/L (IQR)	126 (66.3–267.3)	NA
Median ALP, U/L (IQR)	142 (97–240)	NA
Median total bilirubin, µmol/L (IQR)	12 (8–30)	NA

*Values are no. (%) except as indicated. ALP, alkaline phosphatase; ALT, alanine aminotransferase; IQR, interquartile range; NA, not applicable because all liver function parameters were below the upper limit of reference ranges (Appendix Table 2).

### Results of HCirV Screening in Human Samples

**Figure 1:**
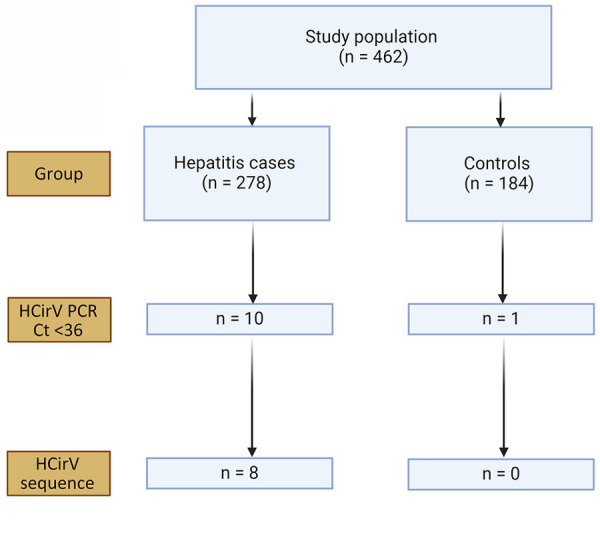
Results of screening in study of human circovirus in patients with hepatitis, Hong Kong. Ct, cycle threshold; HCirV, human circovirus.

The limit-of-detection of the *Rep* gene real-time PCR was 600 copies/mL (Appendix Table 6) with good linearity (Appendix Figure 1). This result was comparable to the limit-of-detection in a recent study (*26*). We screened samples from hepatitis case-patients and from controls for HCirV infection using the *Rep* gene assay, followed by sequencing of positive samples. Of 278 hepatitis patients, 10 (3.6%) tested positive by HCirV PCR, but a sequence could not be obtained from 2 of those. Of the 184 controls, 1 (0.5%) tested positive for HCirV DNA, but a sequence could not be obtained (Figure 1). The 3 samples that tested positive by PCR without an identifiable sequence had high Ct values at >33. We did not regard those as HCirV infections for the purpose of this study.

### HCirV Case Characteristics

**Figure 2:**
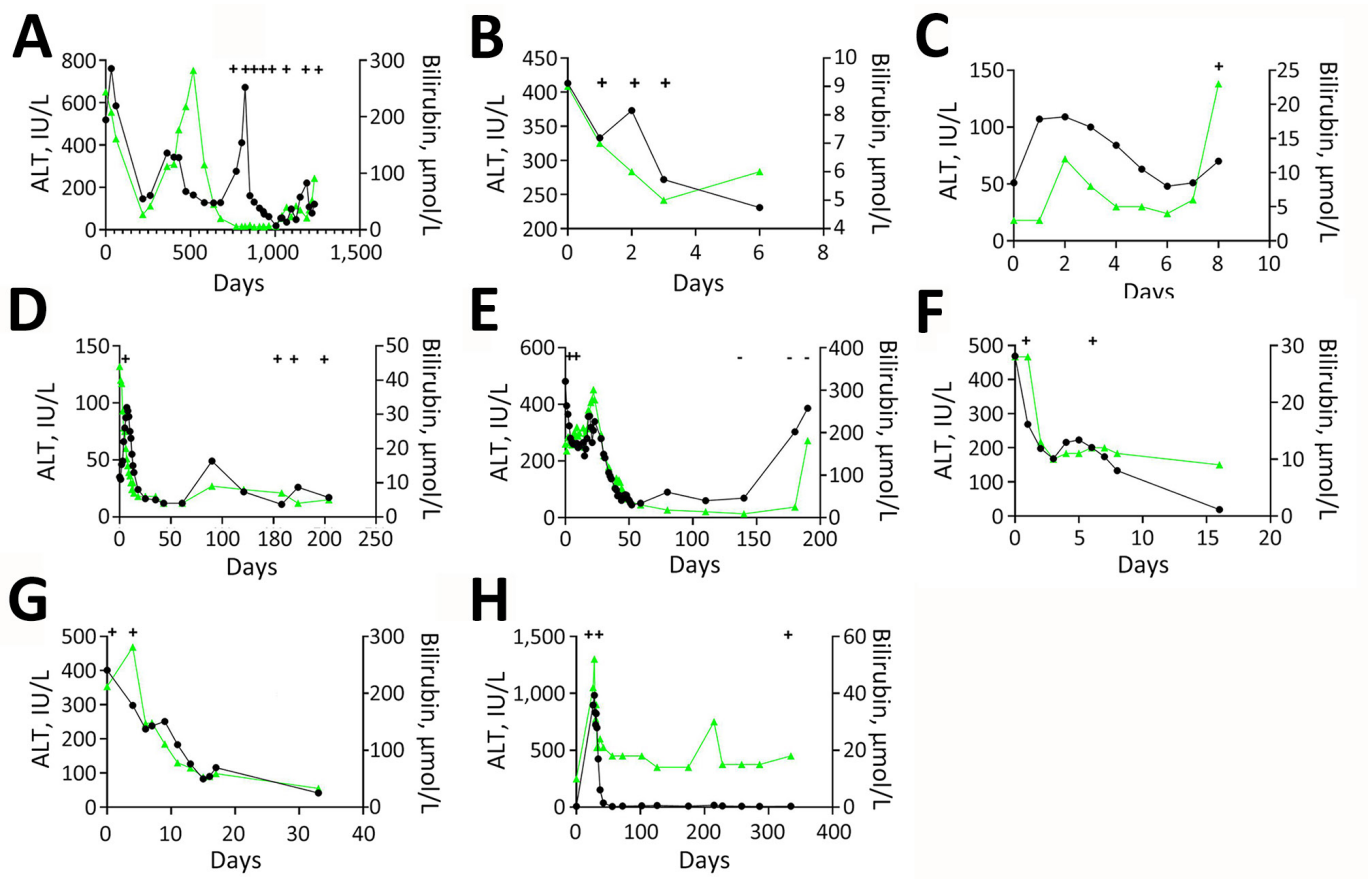
Liver function test kinetics in 8 patients with hepatitis and human circovirus infection, Hong Kong. A) Patient 1; B) patient 2; C) patient 3; D) patient 4; E) patient 5; F) patient 6; G) patient 7; H) patient 8. Black line indicates ALT, and green line indicates bilirubin. Plus signs indicate days when human circovirus DNA was detected in blood, whereas dashes indicate days when human circovirus DNA was not detected in blood. ALT, alanine aminotransferase.

The median age of 8 confirmed HCirV-infected persons (PCR-positive with identifiable sequences) was 47.5 years; 5 were male and 3 female (Table 2). A total of 4 patients had received an organ graft or blood transfusion within 3 months of hepatitis onset, and 4 persons were immunocompromised. None of the patients were known to be infected with HCV or HIV. R factor indicated cholestatic injury in 5 patients, mixed pattern in 2 persons, and hepatocellular injury in 1 person. Consensus on the cause of hepatitis was only available for 1 patient (patient 7); both experts (who were blinded to study objectives and HCirV screening results) agreed with the clinical team on a diagnosis of cholangitis (Appendix Table 7). Investigations conducted for this study identified HEV viremia in 1 patient (patient 3) who had tested negative for HEV IgM (Appendix Table 7). Consensus on the cause of hepatitis in the other 6 patients was lacking. All but 1 patient had acute hepatitis that resolved within a month. HCirV DNA was detectable in plasma or serum of all patients on >1 day during episodes of hepatitis (except for patient 3, who had only 1 blood sample available for testing) (Figure 2). A total of 3 patients died; cause of death was progression of underlying disease or unexplained cardiac arrest rather than hepatitis.

**Table 2:** Clinical and demographic characteristics of patients with human circovirus infection, Hong Kong*

Patient no.	1	2	3	4	5	6	7	8
Age	15	59	39	53	67	75	40	42
Sex	M	M	F	F	M	M	M	F
Immunosuppressive condition	Neuroblastoma	None	None	Kidney graft†	Liver graft†	None	None	Liver graft†
Organs / blood products	Y	N	N	Y	Y	N	N	Y
Peak ALT, U/L	672	413	109	96	532	469	401	983
Peak ALP, U/L	2288	139	174	928	601	274	484	168
R factor	0.6	4.7	1.6	0.3	1.4	3.2	1.6	15.2
Peak bilirubin, µmol/L	91	9	23	44	301	28	281	52
Duration of hepatitis	Chronic	Acute	Acute	Acute	Acute, relapse	Acute	Acute	Acute
Outcome	Death	Survived	Death	Survived	Death	Survived	Survived	Survived
Clinician diagnosis	NIC	NIC	NIC	NIC	Chronic rejection	COVID-19	Cholangitis	Anastomotic stricture
Expert 1 diagnosis	Drug-induced hepatitis	NIC	Ischemic hepatitis	Drug-induced cholestasis	NIC	COVID-19	Cholangitis	Rejection
Expert 2 diagnosis	NIC	NIC	NIC	NIC	NIC	Gallstone	Cholangitis	Anastomotic stricture

*ALP, alkaline phosphatase; ALT, alanine aminotransferase; NIC, no identifiable single cause for hepatitis. †Time from transplantation to hepatitis: patient 4, 1 month; patient 5, 15 years; patient 8, 22 months.

**Figure 3:**
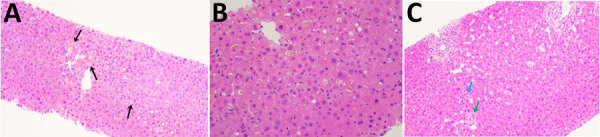
Hematoxylin and eosin stained liver sections of patients in study of human circovirus in patients with hepatitis, Hong Kong. A, B) Cholestasis with bile pigment in zone 3 canaliculi (arrows) in patient 5 at original magnification ×10 (A) and original magnification ×20 (B). C) Acidophil body (blue arrow) with neutrophils around ducts (green arrow) in patient 8; original magnification ×10.

Of the 4 persons with immunosuppressive conditions, 3 were solid organ transplant recipients and1 was receiving chemotherapy for neuroblastoma (patient 1). Patient 1 had relapsing hepatitis episodes over 3 years before eventually dying of the underlying disease (Figure 2). HCirV was detected consistently in all plasma samples over a period of 18 months. Virus loads ranged from 5.65 × 10^3^ copies/mL to 1.75 × 10^6^ copies/mL. Patient 4 (kidney graft recipient) and patient 8 (liver graft recipient) also had persistent HCirV viremia over several months despite resolution of the initial hepatitis episode; viremia was highest during hepatitis (10^4^–10^5^ copies/mL) and declined as LFTs normalized in subsequent months. Archived plasma samples from patient 8 obtained at 1-month and 12-months after transplant tested negative for HCirV DNA, indicating an infection in the second year. Patient 5 (liver graft recipient) had acute hepatitis with transient HCirV DNA detection. Although the hepatitis relapsed around 3 months later, HCirV DNA was no longer detectable in the patient’s plasma. The liver histology of patient 5 during the first hepatitis episode was consistent with cholestasis, whereas histology of patient 8 showed neutrophils around biliary ducts and acidophil bodies with no features of acute cellular rejection (Figure 3). Immunohistochemical staining for HCirV capsid protein on a small liver core biopsy from patients 5 and 8 did not show positive signals (Appendix Figure 2).

For some samples with sufficient volume, we also measured concomitant TTV viral load. HCirV was detected in 2 persons with undetectable TTV viremia. In patient 1, who had serial concomitant measures of HCirV and TTV viral loads, viral loads of HCirV tended to fluctuate much less than those of TTV, suggesting that HCirV is not merely an indirect measure of immunosuppression (Appendix Table 8).

The remaining 2 patients (patients 2 and 6) did not have immunosuppressive conditions. Patient 2, a 59-year-old man with good past health, was admitted with fever for 1 week and a maculopapular skin rash. In addition to hepatitis, blood tests revealed a lymphocytosis (3.8 × 10^9^ cells/L) with occasional atypical lymphocytes. Serologic workup for known causes of infectious mononucleosis including EBV, CMV, and HIV yielded negative results (Appendix Table 7). The peripheral blood EBV viral load was detected at <100 IU/mL. Patient 6 was a 75-year-old man admitted for fever. Blood tests showed parenchymal liver dysfunction (Figure 2). Nasopharyngeal swab samples tested positive for SARS-CoV-2 by reverse transcription PCR with a Ct value of 30.4. Chest radiograph did not show lower respiratory tract involvement, and he did not require supplemental oxygen. Ultrasound showed a gallstone but no cholecystitis or cholangitis.

### HCirV Genome and Phylogenetic Analysis

**Figure 4:**
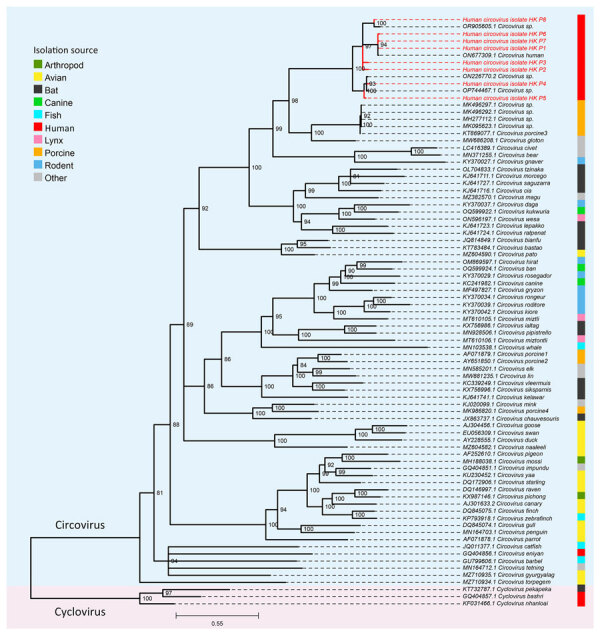
Phylogenetic tree of representative circoviruses and human circoviruses from study of human circovirus in patients with hepatitis, Hong Kong. Maximum-likelihood phylogenetic tree was inferred from a multiple sequence alignment of complete representative *Circovirus* genomes and a mixture of full-length and partial genome sequences from this study (red text) using IQ-TREE (*16*). Three representative species of *Cyclovirus* were used as an outgroup for rooting the tree. Branch supports were assessed using the Shimodaira-Hasegawa–like approximate likelihood ratio test with 10,000 bootstrap replicates. Branches with <80% support were collapsed. Scale bar indicates nucleotide substitutions per site.

Complete HCirV genome was obtained for patient 1 (GenBank accession no. PP968832). Predicted genomic organization of this strain was like other circovirus strains: 2 major open reading frames (ORFs) encoding the *Rep* gene at nucleotide positions (nt) 247–1134 and capsid protein at nt 1368–2012. The *Rep* gene is encoded on the sense strand and the *Cap* gene on the antisense strand. The genome size is 2021 bp, and it is most closely related to the HCirV1 Paris strain (GenBank accession no. ON677309.1), sharing a nucleotide identity of 98.7%. Partial gene sequences were obtained from all the other patients (Appendix Table 9). A phylogenetic tree of complete HCirV genome of patient 1 (HK P1) and partial gene sequences of other isolates showed that strains related to both Paris and Yunnan strains circulate in Hong Kong (Figure 4).

### HCirV Immunoassays

**Figure 5:**
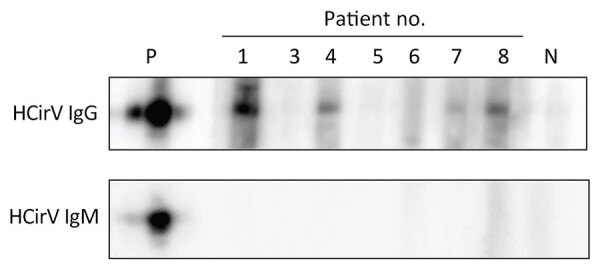
IgM and IgG immunoblots of 7 patients for whom sufficient serum was available from study of HCirV in patients with hepatitis, Hong Kong. P indicates positive control, mouse polyclonal serum raised against HCirV cap protein. N indicates HCirV PCR-negative human donor sample. HCirV, human circovirus.

HCirV Cap protein was expressed in *E. coli*. This peptide showed a band at 25 kDa on SDS-PAGE (Appendix Figure 3, panel A). The peptides spontaneously assembled into VLPs on transmission electron microscopy (Appendix Figure 3, panel B). Serum samples from 7 HCirV-infected persons were available for antibody immunoassays using HCirV VLP-based immunoblots (Figure 5). All patients were negative on IgM immunoblots. Four patients had discernible bands on IgG immunoblots (patients 1, 4, 7, and 8); of those, 3 patients were immunocompromised. As negative controls, we used 9 HCirV PCR-negative donor samples, as well as a serum pool of 900 healthy donors. None of those samples showed bands in the immunoblot, suggesting reasonable specificity (Appendix Figure 3, panel C).

### HCirV Cell-Binding Assay

**Figure 6:**
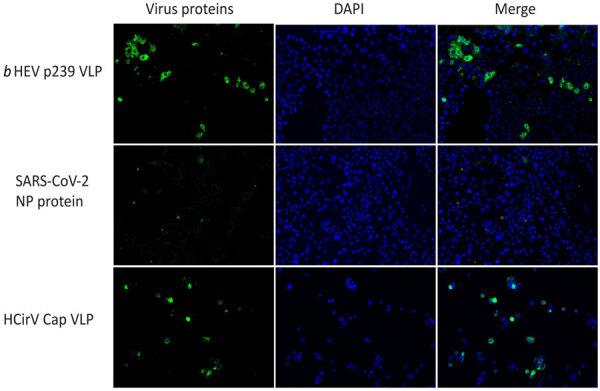
Cell-binding assay showing binding of HCirV VLPs to PLC/PRF/5 cells in study of HCirV in patients with hepatitis. HEV VLPs (*b*HEV p239) is included as a positive control; SARS-CoV-2 NP protein is included as a negative control. Nuclei are counterstained with DAPI. Cap, capsid; HCirV, human circovirus; HEV, hepatitis E virus; NP, nucleoprotein; VLP, virus-like particle.

To further investigate hepatic tropism of HCirV, we tested whether HCirV VLPs could bind to hepatoma cells (PLC/PRF/5). HEV VLPs that are known to bind strongly to PLC/PRF/5 cells served as positive controls. SARS-CoV-2 nucleoprotein served as negative controls. HCirV VLPs were capable of binding to PLC/PRF/5 cells (Figure 6). We found that 27/90 (30%) of cells bound to HCirV VLPs, compared with 30/468 (6.4%) cells that bound to HEV VLPs. We confirmed that HCirV VLPs could bind with Hep-G2, another hepatoma cell line (Appendix Figure 4). Of note, no binding was observed with A549, a lung epithelial cell line (Appendix Figure 4).

## Discussion

The association between HCirV and hepatitis requires careful evaluation. Many human DNA viruses have been classified as orphan viruses despite initial discovery in diseased patient cohorts (*27*–*29*). Prominent examples include anelloviruses and pegiviruses, which were initially believed to be associated with hepatitis but are now generally considered to be nonpathogenic (*30*–*33*).

In this study, we identified HCirV infections in 1.7% of all screened persons using our stringent case definition of PCR positivity with sequenced partial genome. Some persons with high Ct value without identifiable sequences could feasibly have had genuine resolving HCirV infection, in which case the prevalence of HCirV infection in our cohort would be even higher.

Confirming the association between HCirV infection and hepatitis was not straightforward. Alternative explanations for deranged liver function were present in some HCirV patients. However, we could not rule out that HCirV contributed to hepatitis in other cases because of lack of alternate diagnoses, suggestive liver histology, and negative workup for other causes of hepatitis. The case of patient 2, who had an infectious mononucleosis-like syndrome, was particularly striking because of the presence of clinical manifestations that were compatible with acute viral illness. Most infectious mononucleosis-like syndromes are caused by primary/acute EBV, CMV, HIV, and human herpesvirus 6 infections. However, case reports of infectious mononucleosis-like symptoms during noncanonical viral infections such as mpox, parvovirus B19, and human metapneumovirus are also described (*34*–*36*). Therefore, we believe that the infectious mononucleosis-like syndrome in patient 2 was attributable to HCirV. Such clinical manifestations might represent distinctive host responses in a minority of infected patients. We described cholestasis and neutrophil infiltrates in liver tissue of our HCirV-infected patients, which was distinct from the hepatitis with mixed inflammatory infiltrates described by Pérot et al. (*7*) and Hamelin et al. (*37*) in HCirV-infected patients. Those results might represent distinct histopathological findings caused by this virus.

On the basis of the pathobiology of other circoviruses, HCirV human infections could run the gamut from silent infection to well-defined clinical syndromes with complex virus-host interactions determining clinical phenotype (*38*). Detailed surveillance and case descriptions will be required to elucidate the clinical spectrum of this virus. We demonstrate here that HCirV VLPs can bind to liver cells. Tropism for immune cells (like PCV3) could lead to complex virus–host interplay and cytokine dysregulation leading to liver dysfunction (*39*). The role of co-infections and other inflammatory stimuli in HCirV reactivation cannot be excluded. Of the patients reported in this study, 2 had HEV and COVID-19 concomitantly.

In this study, we confirm that HCirV can cause persistent infection. Of note, LFTs can normalize despite ongoing viremia. Because half the HCirV patients identified in this study were immunocompromised, we speculate that HCirV might establish latency and reactivate during immunosuppression. Another possibility is that those patients acquired HCirV through contaminated blood or organ grafts. We were unable to access blood and organ donor data to evaluate this possibility. Furthermore, a large international study involving >200,000 plasma donations did not find evidence of HCirV contamination (*26*). However, on the basis of the relatively higher HCirV detection rate in our study, we cannot rule out that prevalence might be higher in our locality. We speculate whether HCirV might be a previously unidentified porcine circovirus capable of zoonotic transmission. PCV3 has been shown to be transmissible to nonhuman primates through xenotransplantation (*40*).

The first limitation of our study was that we were not able to identify sequences in 3 HCirV PCR-positive samples. Those cases might have been genuine infection cases at early convalescence. The use of rolling circle amplification using a phi29 DNA polymerase would have helped us resolve some of these cases. Other limitations of our study include its retrospective nature; we could not track kinetics of infection in most cases because samples were not available. Liver biopsy was also not available for most cases because of the mild nature of hepatitis. Only small core biopsies of tissue were available from patients 5 and 8, which could explain negative immunohistochemical staining. In situ hybridization would have been a more sensitive approach.

Although not definitive, we believe our findings support further investigation of HCirV as a cause of hepatitis in a proportion of infected human hosts. Larger case–control studies, liver tissue analysis, and infectious disease models are required to investigate this association further. Clinicians should be aware of this new evidence that HCirV could be a cause of hepatitis in some infected individuals.

Appendix Additional information about human circovirus in patients with hepatitis, Hong Kong
