# Supplementary material for: Human Circovirus in Patients with Hepatitis, Hong Kong
Source: Emerg Infect Dis. 2024 Dec;30(12):2521–31. doi: 10.3201/eid3012.241114 (PMC11616632; doi:10.3201/eid3012.241114)
Supplement: Appendix — Additional information about human circovirus in patients with hepatitis, Hong Kong [file 24-1114-Techapp-s1.pdf]

Article DOI: https://doi.org/10.3201/eid3012.241114

EID cannot ensure accessibility for supplementary materials supplied by authors. Readers who have difficulty accessing supplementary content should contact the authors for assistance.

Human Circovirus in Patients with Hepatitis, Hong Kong

Appendix

Supplemental Material

Human circovirus (HCirV) real time PCR

Total nucleic acid was extracted from samples using the EZ1 Virus Mini Kit v2.0 (Qiagen, Hilden, Germany). Real-time PCR assays were performed using QuantiNova Probe PCR Kit (Qiagen) in a LightCycler 480 II Real-Time PCR System (Roche, Basel, Switzerland). Each 20 µl-reaction mix contained 1x QuantiNova Probe PCR Master Mix, 0.4 µM of forward and reverse primers, 0.2 µM probe and 5 µL template DNA. Reactions were incubated at 95°C for 2 min, followed by 45 cycles at 95°C for 5 s and 60°C for 30 s. Quantitation was achieved using plasmid standards prepared using the pCR2.1-TOPO vector (Invitrogen, Carlsbad, USA) cloned with a circovirus replication-associated protein (*Rep*) gene fragment. Plasmid concentrations ranging from 10^2 – 10^6 copies/reaction were used to generate a standard curve for each qPCR run.

Sequencing of human circovirus (HCirV)

HCirV strains were sequenced using Sanger sequencing. Complete genome amplification of human circovirus was performed using primers listed in Appendix Table 2. The PCR mixture (25 µL) contained sample DNA extract, 1X PCR buffer II, 2 mM $MgCl_2$, 200 µM of each dNTP, 1 µM of each forward and reverse primer and 0.625 U of AmpliTaq Gold DNA polymerase (Applied Biosystems, MA, USA). PCR was performed using a thermocycler (Applied Biosystems) with a hot start at 95°C for 10 min, followed by 45 cycles of 94°C for 1 min, 55°C for 1 min, and 72°C for 1 min and a final extension at 72°C for 10 min. The PCR products were

detected by agarose gel electrophoresis and those of correct size were subjected to DNA purification using QIAquick gel extraction kit (QIAGEN). Both strands of the PCR products were sequenced twice with an ABI Prism 3500 Genetic Analyzer (Applied Biosystems) using primers listed in Appendix Table 2. Sequences were assembled and manually edited to produce final sequences of the viral genomes by BioEdit version 7.2.5 (NC State University, Raleigh).

Expression of HCirV cap peptide

The *Cap* gene sequence derived from the YN09/J030 HCirV strain (GenBank accession no.: ON226770) was optimized and cloned into a pET-28a(+) vector (Sangon Biotech, Shanghai, People's Republic of China). Recombinant pET28+-Cap plasmid was transformed into *E. coli* BL21 (DE3) and single colonies were grown in LB medium with 50 g/ml kanamycin at 37°C overnight with shaking at 250 rpm. The culture was diluted 1:20 in 500 ml fresh LB medium and incubated at 37 ◦C at 250 rpm until optical density at 600 nm (OD600) was 0.6–0.8. Isopropyl - d-thiogalactopyranoside (IPTG) was added to a final concentration of 0.5 mM with incubation overnight at 16°C. Cells were harvested by centrifugation at 8000 × g for 10 min at 4 ◦C. The cell pellet was washed in 100 ml of 0.1% Triton in phosphate buffered saline (PBS). After centrifugation, cell pellets were resuspended in 100 mL lysis buffer and sonicated on ice using Soniprep 150. Lysates were divided into supernatant and pellet by centrifugation at 10,000 × g for 20 min at 4°C. Supernatant was collected and purified by His PurNI-NTA Resin (Thermo Fisher Scientific, Waltham, USA). Expression and solubility of Cap protein was analyzed by sodium dodecyl sulfate polyacrylamide gel electrophoresis (SDS-PAGE) and Coomassie blue staining on a 15% polyacrylamide gel.

Soluble Cap protein was purified by ENrich SEC 650 10x300 column (Bio Red NGC chromatography system). Protein samples were separated at a flow rate of 0.5 ml/min. Fraction aliquots were analyzed by 12% SDS-PAGE and Western blotting. Protein concentrations were determined by Bradford assay (Bio-Rad, Hercules, USA) using bovine serum albumin (BSA) as a standard. Fractions containing purified protein were pooled and stored at 4°C.

Transmission electron microscopy

Cap protein was ultracentrifuged using Amicon Ultra-0.5 tubes (Merck, New Jersey, USA) according to manufacturer instructions. The filtrate was adsorbed onto a glow-discharged

formvar/carbon support grids for 5 minutes, then stained with 3% phosphotungstic acid for 30 seconds. Grids were examined using a transmission electron microscope (Philips CM100).

Polyvalent murine anti-cap antisera

Cap protein (10 ug) was mixed with an equal volume of QuickAntibody adjuvant (Biodragon Immunotechnologies, Beijing, People's Republic of China; Cat# KX0210042) in advance. Six-week-old female BALB/c mice (n = 2) were immunized intramuscularly with the protein, and then boosted intramuscularly at day 14. On day 38, blood was harvested and spun down to separate serum, which served as controls for the immunoblot experiments.

HCirV IgM and IgG immunoblots

HCirV cap proteins separated by SDS-PAGE were transferred onto a nitrocellulose membrane (Immobilon 0.45 µm; Merck Millipore, Burlington, USA). After blocking 10% skim milk in 0.3% TPBS, the membrane was incubated with different antibodies/ sera lane-by-lane using the Mini-PROTEAN II Multiscreen Apparatus (BIO-RAD) at room temperature for 1 h (human sera (1:100) or mouse sera (1:2000)). The blots were then washed in 0.3% TPBS and exposed to horseradish peroxidase (HRP)-conjugated secondary antibodies. After washing, membranes were visualized using the Luminescent image analyzer (GE Healthcare, Chicago, USA).

Analytical characteristics of the HCirV PCR assay

The limit of detection (LOD) of the HCirV real-time PCR assay was evaluated using 2-fold serial dilutions of a sample extract or a plasmid containing the target insert ranging from 1.25 copies/reaction to 40 copies/reaction. Each concentration was tested in 8 replicates (Appendix Table 5). The LOD of the circovirus real-time PCR assays targeting *Cap* and *Rep* gene regions were 5 copies/reaction and 10 copies/reaction, respectively. Both assays did not cross-react with samples containing HAV, HBV, HCV, HEV, herpes simplex virus (HSV-1), HSV-2, varicella zoster virus (VZV), CMV, EBV, HHV-6, and enterovirus. Linearity of the *Rep* gene assay used for screening human samples was evaluated by analyzing a dilution series of the plasmid with various concentrations (Appendix Figure 1). Circovirus DNA quantitation by the in-house circovirus qPCR assay was linear (R^2 = 0.9989) across approximately eight orders of magnitude, ranging from 10 to 10^8 copies/reaction. Therefore, the analytical characteristics of HCirV real-time PCR assays was considered sufficient to screen samples.

References

1. Pérot P, Fourgeaud J, Rouzaud C, Regnault B, Da Rocha N, Fontaine H, et al. Circovirus hepatitis infection in heart-lung transplant patient, France. Emerg Infect Dis. 2023;29:286–93. PubMed https://doi.org/10.3201/eid2902.221468

Appendix Table 1. Upper limits of reference ranges for liver function tests used in this study

Liver function test	Male	Female
Alkaline phosphatase (U/L)	110	93
Alanine aminotransferase (U/L)	58	36
Aspartate aminotransferase (U/L)	38	30
Total bilirubin (µmol/L)	23	23
Gamma-glutamyl transpeptidase (U/L)	62	35

Appendix Table 2. Primers and probes for human circovirus detection used in this study*

Assay	Gene target	Primer / probe sequence (5′- 3′)		Product size (bp)
Human sample screening	*Rep*	Forward	GTGTTTTACGATMAACAACTGGAC	145 bp
		Reverse	GTTCTTGAAATTCACGTAYCCTTG	
		Probe	HEX- TATCGGCARAGAGGTTGGAA-IABkFQ	

*bp, base pairs.

Appendix Table 3. Primers used for Sanger sequencing of human circovirus*

Primer name	Sequence (5′ - 3′)	Position
HcirV-YN-F1	CTGGAACCAGGAGGAAGATTGAG	25–47
HCirV1_101F	GAGCAAGGTGCTGTATGGTG	101–120
HCirV1-F3	GTGTTTTACGATMAACAACTGGAC	276–299
HCirV1-R3	GTTCTTGAAATTCACGTAYCCTTG	397–420
HCirV1_rep-330F	TGGCAAAGAGAAAGCGAAGTA	330–350
HcirV-YN-R1	TGTTCTTGAAATTCACGTATCCTTGC	396–421
HCirV1_rep-555R	GCCACTCTCTCCAATCTCCA	536–555
HCirV1_rep-902R	TCATCATAAGGAACCCACCCA	882–902
HCirV1-F2	GATGGCTATGATGGGGAGG	838–856
HCirV1-R2	CCCCTTAACAGGAACCTTCA	932–951
HCirV1_rep-882F	TGGGTGGGTTCCTTATGATGA	882–902
HCirV1_capsid-1562R	CCTCTCATCCATCTTACTGGCA	1541–1562
HCirV1-Rv1†	AGAGTTCCACCAGGTTCTGC	1484–1504
HCirV1-Fw1†	ACCTGGATGGACCCTGGAAT	1659–1678
HCirV1_capsid-1590F	TCTGTTAGCCTTCCAAAGTCTG	1590–1611
HCirV1_rep-350R	TACTTCGCTTTCTCTTTGCCA	330–350

*Primer position corresponding to the genome sequence of Human circovirus 1 strain Paris (GenBank: ON677309.1). F indicates forward primer; R indicates reverse primer.
†Primers designed by Pérot et al. (*1*).

Appendix Table 4. Methodology of testing of agents of viral hepatitis

Pathogen	Assay (manufacturer)
Hepatitis A virus	Vidas anti-HAV IgM (bioMérieux, Marcy-l'Étoile, France)
Hepatitis B virus	Alinity HBsAg (Abbott, Chicago, USA)
	Alinity Anti-HBc (Abbott)
	Cobas 4800 System HBV DNA (Roche, Basel, Switzerland)
Hepatitis C virus	Anti-HCV (Abbott)
Hepatitis E virus	HEV IgM (Wantai, Beijing, People's Republic of China)
	In-house developed real-time RT-PCRs targeting *Paslahepevirus balayani* and *Rocahepevirus ratti*
Epstein-Barr virus	In-house developed real-time PCR assay
Human cytomegalovirus (CMV)	Vidas anti-CMV IgM (bioMérieux)
	In-house developed pp65 antigen or real-time PCR assay
Human herpesvirus 6	In-house developed real-time PCR assay
Human herpesvirus 7	In-house developed real-time PCR assay
Adenovirus	In-house developed real-time PCR assay
Enterovirus	In-house developed real-time RT-PCR assay

Appendix Table 5. Causes of hepatitis in patients with hepatitis of known etiology (n = 123), Hong Kong Special Administrative Region, People's Republic of China

Cause of hepatitis	Number (%)
Metabolic dysfunction-associated steatotic liver disease	11 (8.9)
Gallstone disease / acute cholangitis / liver abscess	17 (13.8)
Alcohol-related liver disease	3 (2.4)
Drug-induced liver injury	22 (17.9)
Acute cellular rejection of graft liver	7 (5.7)
Autoimmune hepatitis	2 (1.6)
Graft versus host disease	11 (8.9)
Malignancy	7 (5.7)
Viral hepatitis	23 (18.7)
HAV	1
HBV	13
HCV	4
HEV	5
Systemic causes (including shock, post-cardiac arrest, right-sided heart failure, thyrotoxic hepatitis, systemic infections)	17 (13.8)
Acute pancreatitis	2 (1.6)
Polycystic liver disease	1 (0.8)

Appendix Table 6. Determining limits of detection (LOD) of the human circovirus PCR assay used in this study*

	Result							
Concentration (copies/reaction)	Test 1	Test 2	Test 3	Test 4	Test 5	Test 6	Test 7	Test 8
Rep gene assay								
40	+	+	+	+	+	+	+	+
20	+	+	+	+	+	+	+	+
10	+	+	+	+	+	+	+	+
5	–	+	+	+	+	–	–	–
2.5	–	+	–	+	+	+	–	+
1.25	–	+	–	–	–	+	+	–
NTC	–	–	–	–	–	–	–	–

*NTC, no template control; +, detected; –, not detected.

Appendix Table 7. Investigations in patients with human circovirus infection, Hong Kong Special Administrative Region, People's Republic of China*

Patient	1	2	3	4	5	6	7	8
HAV IgM	N	N	N	N	N	N	N	N
HBsAg	N	N	N	N	N	N	N	N
Anti-HBc	N	N	N	P	N	P	N	N
HBV DNA	ND	ND	ND	N	ND	N	ND	ND
Anti-HCV	N	N	N	N	N	N	N	N
HEV IgM	N	ND	N	N	N	N	N	N
HEV RNA	N	N	**Detected**	N	N	N	N	N
HIV	N	N	N	N	N	ND	ND	N
EBV VCA IgM	N	N	N	N	ND	N	N	N
EBV DNA	<100 IU/mL	<100 IU/mL	N	N	<100 IU/mL	N	N	N
CMV IgM	N	N	ND	ND	ND	N	N	N
CMV DNA / CMV pp65 Ag	N	N	N	N	N	N	N	N
HHV-6 DNA	N	N	N	N	N	N	N	N
HHV-7 DNA	N	N	N	N	N	N	N	N
Adenovirus DNA	N	N	N	N	N	N	N	N
Enterovirus RNA	N	N	N	N	N	N	N	N
ANA	N	N	ND	N	N	N	ND	ND
Anti-mitochondrial Ab	N	N	ND	N	N	N	ND	ND
ANCA	N	N	ND	N	N	ND	ND	ND
Ultrasound imaging	Normal liver. Non-specific hypoenhancing lesions in both liver lobes	Normal liver. Biliary ducts are not dilated.	ND	Normal liver echotexture. No intrahepatic duct dilatation	Mildly coarsened parenchymal echogenicity Biliary ducts are not dilated.	Normal liver Gallstone, no gallbladder wall thickening.	Dilated common bile duct and intrahepatic ducts with intraductal stone in distal common bile duct.	No gross intrahepatic lesions. Intrahepatic ducts are not dilated.
Liver biopsy		ND	ND	ND		ND	ND	

*CMV, cytomegalovirus; EBV, Epstein-Barr virus; HHV-6, human herpesvirus 6; HHV-7, human herpesvirus 7; N, not detected; ND, not done; P, detected.

Appendix Table 8. Human circovirus (HCirV) and TT virus loads in peripheral blood of HCirV-infected patients, Hong Kong Special Administrative Region, People's Republic of China. Days of sample collection follows that depicted by + in Figure 2 of the main manuscript.

Patient	HCirV load (log copies/ml)	TT virus load (log copies/ml)
P1	5.18	6.66
	5.20	6.88
	4.63	6.57
	4.5	5.02
	3.75	4.39
	4.33	5.42
	5.59	N/A
	5.43	N/A
	5.21	N/A
	5.32	8.37
	4.73	8.27
P2	3.78	N/A
	3.49	N/A
	3.48	N/A
P3	5.04	0
P4	5.59	N/A
	4.02	0
	3.83	N/A
	3.90	N/A
P5	4.89	N/A
	3.71	3.86
P6	4.86	N/A
	3.44	5.32
P7	3.51	5.71
	3.82	N/A
P8	2.78	3.62
	4.13	N/A
	2.78	5.03
N/A: not available		

Appendix Table 9. Genome positions of available sequences from HCirV patients, Hong Kong Special Administrative Region, People's Republic of China. Genome positions labeled with reference to ON677309.1. Patient numbering as per Table 2.

Patient no.	Genome position (bp)
P1	1–2021
P2	300–396
P3	276–417
P4	14–879
P5	146–387
P6	276–421
P7	65–388
P8	25–722

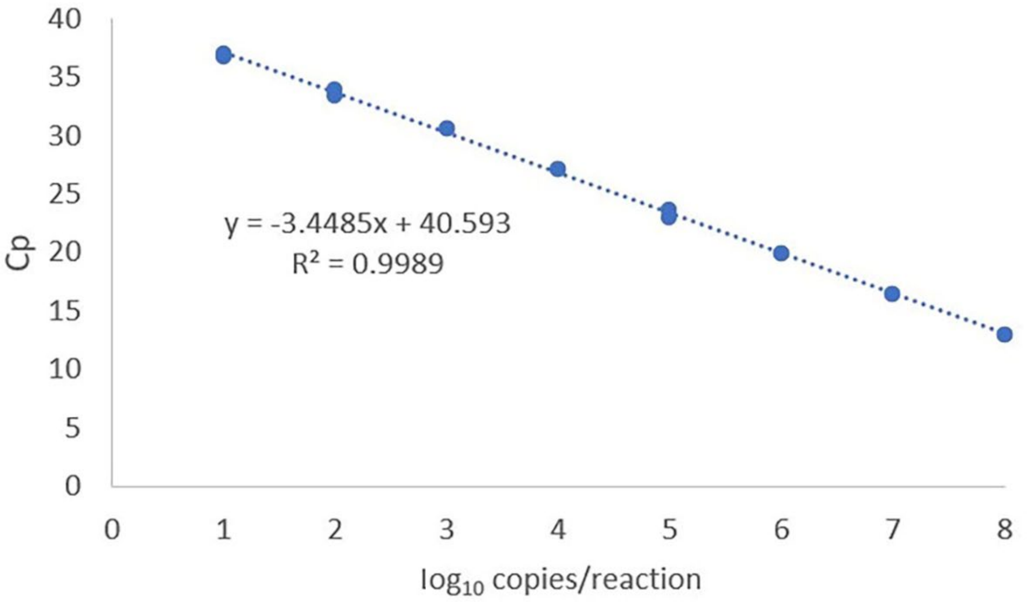

Appendix Figure 1. Linear regression of the *Rep* gene real-time PCR assay using a dilution series of the plasmid containing the HCirV1 *Rep* gene fragment.

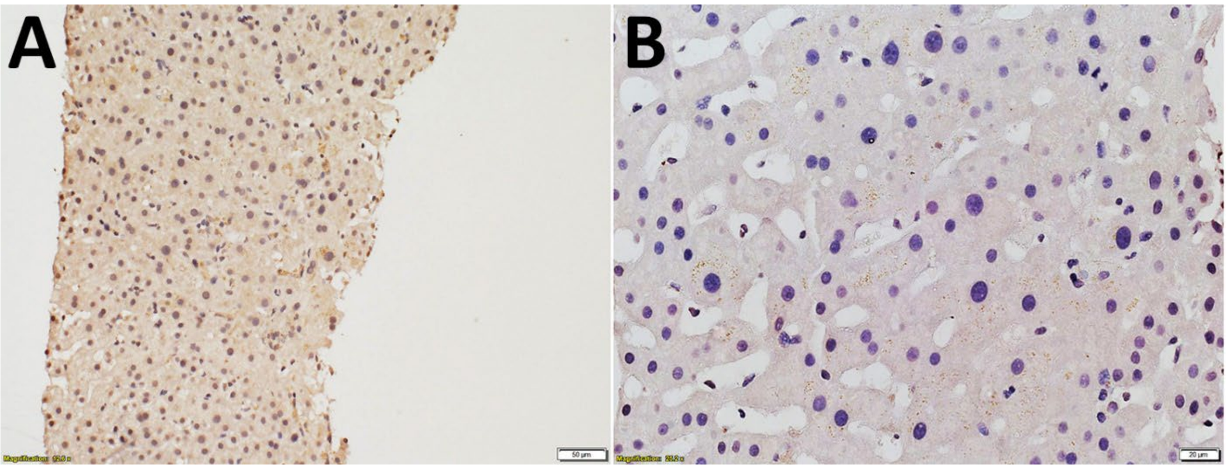

Appendix Figure 2. Immunohistochemical staining of liver tissue from (A) patient 5 and (B) patient 8.

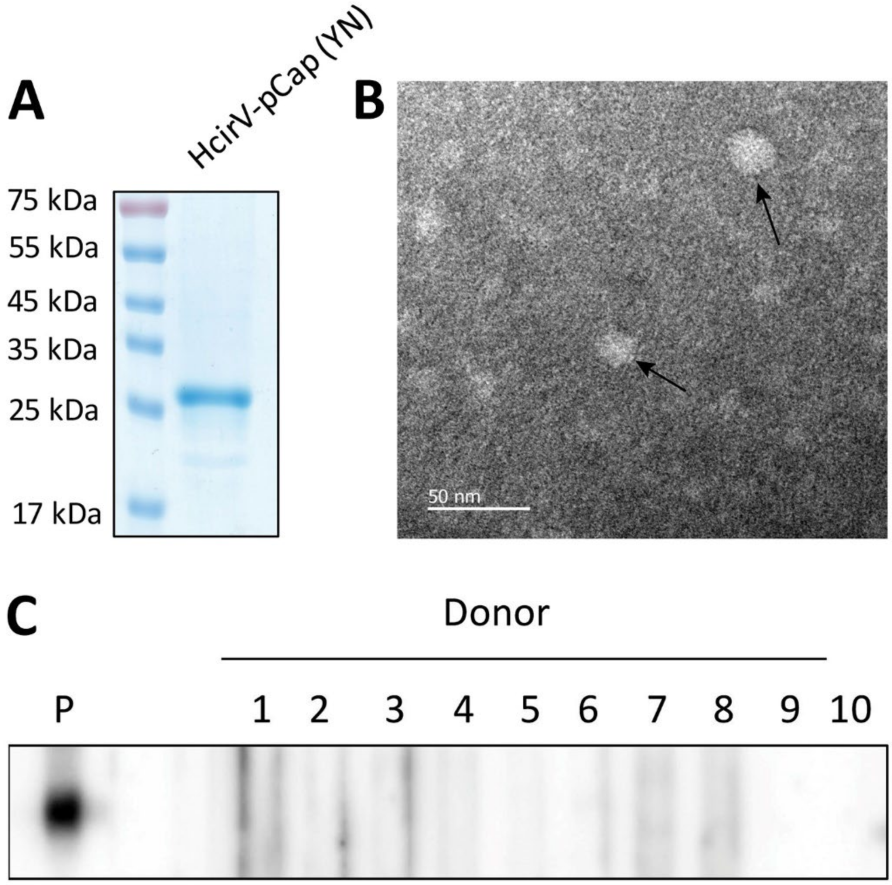

Appendix Figure 3. (A) SDS-PAGE of HCirV Cap protein. (B) Transmission electron microscope image of HCirV Cap protein showing assembly into VLPs. (C) IgG immunoblot of HCirV PCR negative donor samples: lanes 1 – 9 represent individual donor sera while lane 10 is a pooled donor sample

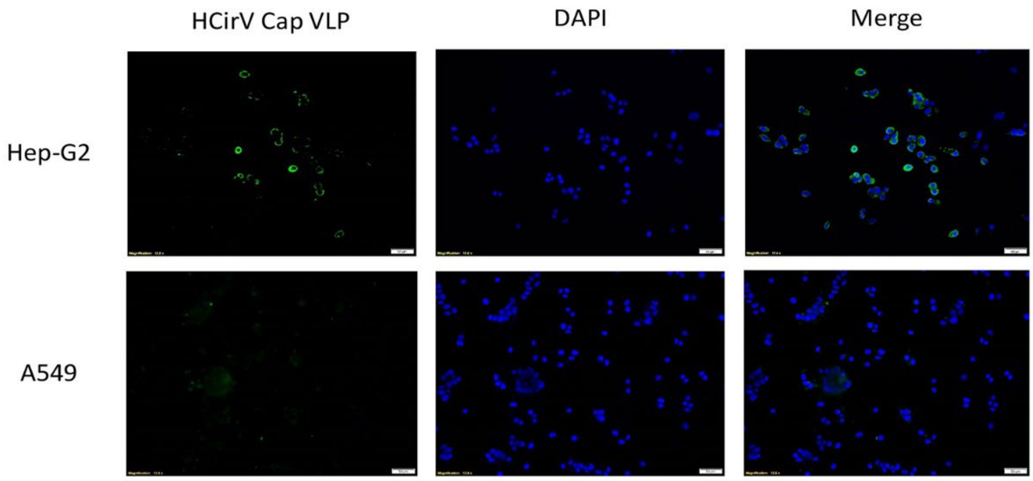

Appendix Figure 4. Cell binding assay showing binding of human circovirus virus-like particles (VLPs) to Hep-G2 cells and A549 cells. Nuclei are counterstained with DAPI.
